# Examination of the vessel's shape, resistive force and volumetric-aqueous efficiencies to optimize the vessels' foil under noise propagation

**DOI:** 10.1016/j.heliyon.2024.e29634

**Published:** 2024-04-16

**Authors:** Zhiheng Xu, Yan Shi, Shelesh Krishna Saraswat, Dheyaa J. Jasim, Ahmad Keshavarzi, Soheil Salahshour, Ahmed Alawadi, S.A. Eftekhari

**Affiliations:** a Yangzhou Polytechnic Institute, Yangzhou, 225000, Jangsu, China; b Department of Mechanical Engineering, Hebei University of Water Resources and Electric Engineering, Cangzhou, 061001, Hebei, China; c Industrial Manipulator Control and Reliability Technology Innovation Center of Hebei, Cangzho, 061001, Hebei, China; d Department of Electronics and Communication Engineering, GLA University, Mathura, 281406, India; e Department of Petroleum Engineering, Al-Amarah University College, Maysan, Iraq; f Department of Mechanical Engineering, Khomeinishahr Branch, Islamic Azad University, Khomeinishahr, Iran; g Faculty of Engineering and Natural Sciences, Istanbul Okan University, Istanbul, Turkey; h Faculty of Engineering and Natural Sciences, Bahcesehir University, Istanbul, Turkey; i Department of Computer Science and Mathematics, Lebanese American University, Beirut, Lebanon; j College of Technical Engineering, The Islamic University, Najaf, Iraq

**Keywords:** Hull foil, Optimization, SST k-ω turbulence model, Resistive force and volumetric-aqueous efficiency, Noise propagation

## Abstract

There are several parameters in designing undersurface vessel forms, the most important of which is the hull's total strength, which includes the strength of the hull and its attachments. According to studies, 70 % of the total strength of the vessels is related to their hull only without attachments. The hull has three major parts: nose, cylinder, and heel. The advanced vessels' architecture has a parallel shape (cylinder shape). This cylindrical part is important in examining the used volume by pilots and vessel equipment. This paper uses the CFD method to examine the vessel's shape, and the resistive force and volumetric-aqueous efficiencies are extracted. An optimum profile is extracted by the values of resistive force and volumetric-aqueous efficiencies. The results indicate the significant effect of the hull form on the hydro-acoustic noise of the hull. In other words, by optimizing the hydrodynamic form of the hull, the noise propagation can be reduced as much as possible. Also, the linear slope of the optimized hull is not optimized more than the hull. This means that the turbulence caused by the optimized hull has a higher damping potential.

## 1 Introduction

Over the past decades, the use of vessels has been considered in various fields such as military, oceanography, the environment, etc. Estimating the vessel's hydrodynamic coefficients is very important at the design stage. The design optimization process includes determining the dimensions and shape of the body, hydrodynamics and speed control, equipment selection, structural design, stability control, control, and cost control [[1], [2], [3], [4]]. Today, the computational fluid dynamics (CFD) method is used as a powerful assessment tool in all areas of vessel design. CFD is a branch of fluid mechanics that uses numerical methods and algorithms to analyze and analyze problems involving airflow [5]. Using the CFD technique, all flow field variables can be fully described. A large number of fluid flow processes can be studied simultaneously; therefore, completely complex problems can be simulated using the CFD technique. Simulation using the CFD technique has the advantage that valuable information about flow behavior can be obtained without experimentation. So far, a lot of research has been done to optimize the vessels. Some examples include the optimization of the vessels' hull shape for the best velocity, reducing the vortices created by the vessel, and optimizing the equipment [6,7]. Vessel stability and control are the most important parameters of optimal design that are strongly influenced by the hydrodynamic forces applied to the vessel [8]. Hydrodynamic forces are expressed as mathematical equations in hydrodynamic coefficients [8]. Without the exact knowledge of the hydrodynamic coefficients of a vessel, doing hydrodynamic analyses and studying the dynamics of the motion of several degrees of freedom of these bodies is not possible. The interactive effects of hull movement and the environment are the function of its geometric profile, velocity and type of maneuver, material, and type of applied fluid regime [9]. Hence, the recognition and accurate estimation of these forces are essential for improving undersurface vessels' performance and proper control. Many researchers studied the behavior of vessels with the flow of fluid and calculated the forces acting on them. For example, Honaryar et al. [10] investigated the effect of the geometric shape of the tail on the drag and lifted forces and maneuverability of the submersible automatic robot. The results indicate that the tail length and tail cone angle increase and decrease maneuverability, respectively. Saout et al. [11] performed a hydrodynamic analysis of the intelligent submarine floating near the free surface and evaluated the dynamic stability. Their floating body shape differs from conventional bodies in that it moves close to the free surface, especially in the nose and tail. In another study, Phillips et al. [12] used CFD to design cost-effective hydrodynamics for intelligent submarine vessels. In his work, the effect of body shape on the resistance-velocity curve of the float was investigated. Zhang et al. [13] simulated the flow around symmetrical bodies of intelligent submarines floating in steady rotation. In their method, CFD was performed based on RANS equations and numerical simulations were performed for different body shapes such as ellipses with length-to-diameter ratios of 4 and 6. Several other forms were compared with the results of laboratory tests. Jefferson et al. [14] performed numerical methods to compute the coefficients and forces on the airship. This study shows that validation using experimental results from wind tunnels is difficult. Still, this solution will be acceptable for the initial phases and initial design. Moonesun et al. [15,16] used CFD to analyze the shape of a submarine heel. They examined several samples of heel form with different geometric equations and suggested several samples of heel form from the point of view of minimum strength. In their study, the dimensions of amplitude, diameter, nose shape, and overall length are constant. Suastika et al. [17] designed a foil system and retrofitted it to a catamaran to reduce its total resistance. Free surface effects were modeled, i.e., the generation of waves due to the vessel's movement on the water surface. The results show that total resistance, reaching a value of approximately 11 %, was increased at relatively low speeds due to the foil system. However, at higher speeds, the foil system decreases the total resistance. Doyle et al. [18] investigated the effects of different sail edges on the submarine flow field and hydrodynamic noise to reduce the noise produced by the sail. They compared the results with the experimental data, which show the accuracy and good performance of the numerical method. The results show that the hydrodynamic noise of submarines can be effectively suppressed by changing the leading edge of the sail, and the sound pressure level can be dropped by 4.69 dB.

According to the presented articles, due to the great importance of studying the behavior of submarines against the current, many people analyzed the dynamic and hydrodynamic geometric shapes of the tail and heel. In this research, the flow optimization is initially performed, and in the next stage, the noise propagation is focused. Examining the flow around the submarine with all its attachments creates a wide and important field of activity for CFD. Therefore, the CFD method was used to investigate the shape of the container the resistance force, and volumetric-aqueous efficiency. This study examines the effect of various parameters such as the diameter of the cylindrical piece and the effect of the nose and heel curvature. Also, for a more comprehensive examination, the effect of all amplitudes of the frequency spectrum is investigated.

## 2 Problem geometry

The aerodynamic or hydrodynamic object has its unique profile, following the special mathematical functions that various researchers obtained. Therefore, because the purpose of this work is to obtain the optimal SUBOFF vessel's profile by CFD, first, the main profile in Refs. [1,19] is presented here.

### 2.1 Hull form equations

Eq. (1) is used to define the nose curve of the SUBOFF model 0Ft≤x≤3.333333Ft , where $R_{\max}$ is the maximum vessel radius.

(1) $$R=R_{\max}{\left\{\begin{array}{c} 1.126395101x{\left(0.3x-1\right)}^{4}\\ +0.442874707x^{2}{\left(0.3x-1\right)}^{3}\\ +1-{\left(0.3x-1\right)}^{4}\left(1.2x-1\right) \end{array}\right\}}^{\frac{1}{2.1}}$$

$$R_{\max}=\frac{5}{6}\text{ft}$$

The central hull equation is valid for 3.333333Ft≤x≤10.645833Ft.

(2) $$R=R_{\max}$$

The equation for the rear part of the hull that is valid for 10.645833ft≤x≤1397167ft begins after the central hull and is as follows:

(3) $$R=R_{\max}{\left\{\begin{array}{c} r_{h}^{2}+r_{h}k_{0}\zeta^{2}\\ +\left(20-20r_{h}^{2}-4r_{h}k_{0}-\frac{1}{3}k_{1}\right)\zeta^{3}\\ +\left(45-45r_{h}^{2}-6r_{h}k_{0}-k_{1}\right)\zeta^{4}\\ +\left(36-36r_{h}^{2}-4r_{h}k_{0}-k_{1}\right)\zeta^{5}\\ +\left(-10+r_{h}^{2}{+}_{h}k_{0}-\frac{1}{3}k_{1}\right)\zeta^{6} \end{array}\right\}}^{\frac{1}{2}}$$

$$r_{h}=0.1175,k_{0}=10,k_{1}=44.6244$$

$$\zeta =\frac{13.979167-x}{3.333333},x\;\text{in}\;\text{feet}$$

and for the cone region 13.97167ft≤x≤14.291667ft , the equation is as follows.

(4) $$R=0.1175\;R_{\max}\;{\left[1-{\left(3.2x-44.733333\right)}^{2}\right]}^{\frac{1}{2}}$$

**Fig. 1:**
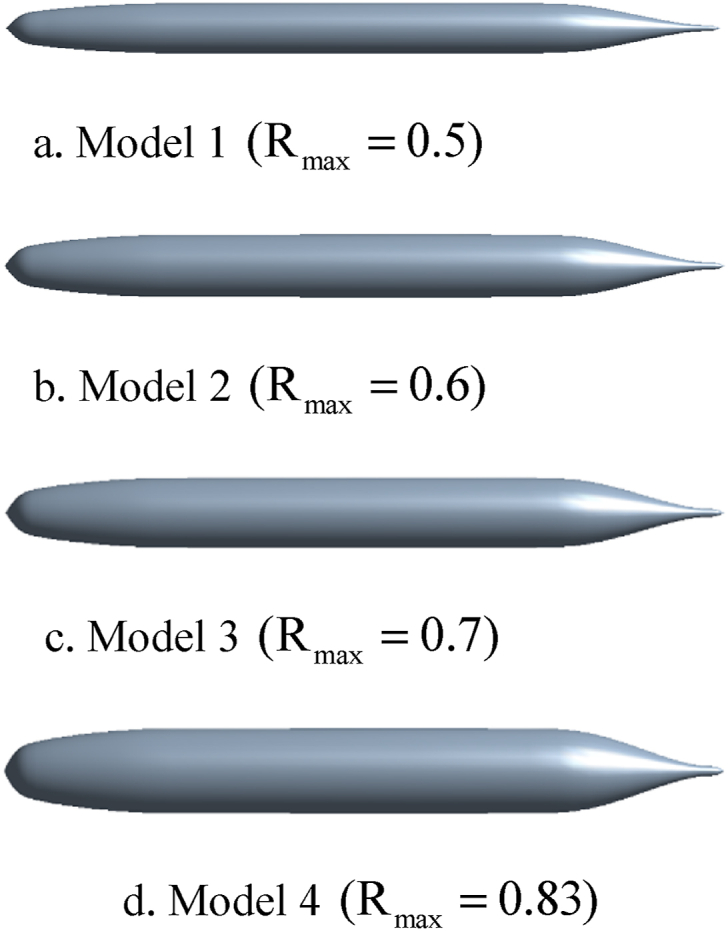
The various profiles.

Fig. 1 shows the various profiles obtained from the SUBOFF equations by changing the coefficient.

### 2.2 Equations of the shapes of the nose and heel

**Fig. 2:**
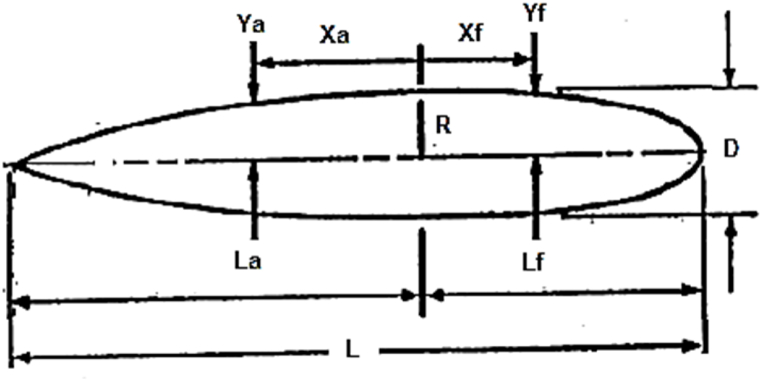
Parameters and coordinate system for Eqs. (5), (6)) to determine the vessel hull.

Different equations are used to define the nose and heel of the advanced vessels [20,21]. The same equations are used to define the nose and heel. The nose equation is as follows. Fig. 2 shows the parameters and coordinate system to determine the vessel hull and the heel equation is as follows.

(5) $$Y_{f}=R{\left(1-{\left(\frac{X_{f}}{L_{f}}\right)}^{n_{f}}\right)}^{\frac{1}{n_{f}}}$$

(6) $$Y_{a}=R{(1-\left(\frac{X_{a}}{L_{a}}\right)}^{n_{a}})$$

**Fig. 3:**
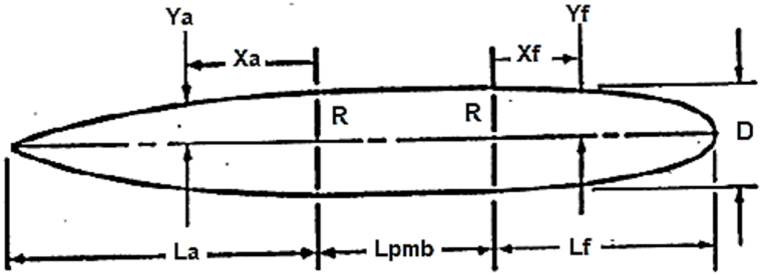
Parameters and the coordinate system for Eqs. (7), (8)

This profile lacks middle length; thus, it should be reformed into Eqs. (7), (8). In these equations, with the addition of the $L_{c}$ constant, the length of the cylindrical part is also added to the profile. Fig. 3 is presented to understand this issue better. The heel equation to the middle cylindrical part is as follows,

(7) $$r_{a}=R{(1-\left(\frac{\left(L_{a}-x\right)}{L_{a}}\right)}^{n_{f}})$$

The nose equation to the middle cylindrical part is as follows,

(8) $$r_{a}=R\left(1-{\left(\frac{\left(L_{a}-X\right)}{L_{a}}\right)}^{n_{f}}\right)$$

### 2.3 Governing equations

For modeling, the k-ω-Wilcox model [12] is used regionally for areas near solid walls and uses the standard k-Epsilon model (in the formulation of k-ω) near the boundary layer and in tension-free layers. Eddy viscosity is defined by the turbulent kinetic energy function (k) and the turbulence's specific loss rate or frequency (ω) equation,

(9) $$\mu_{t}=\frac{\rho k/\omega }{\max\left[1;\Omega F_{2}/\left(a_{1}\omega \right)\right]}\;a_{1}=0.31$$

The maximum eddy viscosity in the turbulent boundary layers is limited by forcing the shear stress to turbulent kinetic energy (a_1_). This effect was created using the auxiliary function F_2_ and the absolute value of vorticity (ω). The auxiliary function F_2_ is defined as the distance from the wall (y).

(10) $$F_{2}=\left\{{\left(\max\left[2\frac{\sqrt{k}}{0.09\omega y};\frac{500\mu }{\rho y^{2}\omega }\right]\right)}^{2}\right\}$$

Two equations of the model are defined as follows.

(11) $$\frac{\partial \rho k}{\partial t}+\frac{\partial }{\partial x_{j}}\left(\rho u_{j}k-\left(\mu +\sigma_{k}\mu_{t}\right)\frac{\partial k}{\partial x_{j}}\right)=\tau_{t_{\text{ij}}}S_{\text{ij}}-\beta^{*}\rho \omega k$$

(12) $$\frac{\partial \rho \omega }{\partial t}+\frac{\partial }{\partial x_{j}}\left(\rho u_{j}\omega -\left(\mu +\sigma_{\omega }\mu_{t}\right)\frac{\partial \omega }{\partial x_{j}}\right)=P_{\omega }-\beta \rho \omega^{2}+2\left(1-F_{1}\right)\frac{\rho \sigma_{\omega 2}}{\omega }\frac{\partial k}{\partial x_{j}}\frac{\partial \omega }{\partial x_{j}}$$

The ω production is sometimes approximated with the absolute value of vorticity.

(13) $$P_{\omega }\equiv 2\gamma \rho \left(\delta_{\text{ij}}-\omega \delta_{\text{nn}}\delta_{\text{ij}}/3\right)\delta_{\text{ij}}\approx \gamma \rho \Omega^{2}$$

In this study, they are considered for the vessel foil by turbulence SST k- ω and FW-H hydro-acoustic methods simultaneously. The FW-H equation is a non-homogeneous wave equation. This equation is obtained by manipulating the continuity and Navier-Stokes equations. The FW-H equation is written as follows:

(14) $$\frac{1}{a_{0}^{2}}\frac{\partial^{2}p^{\prime }}{\partial t^{2}}-\nabla^{2}p^{\prime }=\frac{\partial^{2}}{\partial x_{i}\partial x_{j}}\left\{T_{\text{ij}}H\left(f\right)\right\}-\frac{\partial }{\partial x_{i}}\left\{\left(P_{\text{ij}}n_{j}+\rho u_{i}\left(u_{n}-v_{n}\right)\right)\delta \left(f\right)\right\}+\frac{\partial }{\partial t}\left\{\left(\rho_{0}v_{i}+\rho \left(u_{n}-v_{n}\right)\right)\delta \left(f\right)\right\}$$

where $u_{i}$ is the velocity component of the fluid in $x_{i}$ direction. $u_{n}$ is the velocity component of the fluid in the direction perpendicular to the object's surface. Functions H(f) and δ(f) are the step and the Dirac delta functions. It should be noted that f=0 determines the location of the object's surface. $p^{\prime }$ is the sound pressure, $n_{j}$ is the normal vector, $a_{0}$ is the sound velocity at far distances of the fluid, and $T_{\text{ij}}$ is Light Hill stress tensor defined as follows:

(15) $$T_{\text{ij}}=\rho u_{i}u_{j}+P_{\text{ij}}-a_{0}^{2}\left(\rho -\rho_{0}\right)\delta_{\text{ij}}$$

$P_{\text{ij}}$ is the compressive stress tensor, which is defined for the desired fluid as follows:

(16) $$P_{\text{ij}}=p\delta_{\text{ij}}-\mu \left[\frac{\partial u_{i}}{\partial x_{j}}+\frac{\partial u_{j}}{\partial x_{i}}-\frac{2}{3}\frac{\partial u_{k}}{\partial x_{k}}\delta_{\text{ij}}\right]$$

The free flow variables were shown with an index of 0. Only by assuming acoustic free flow and that there is no obstacle between the acoustic source and receiver location can it be integrated analytically. The full solution consists of surface and volume integrals. Surface integrals represent polar and bipolar acoustic sources and partially the four-pole sources, while volumetric integrals represent four-pole sources in the range outside the source levels. The volume or proportion of volumetric integrals is negligible when the fluid is incompressible (the Mach number is less than 0.3) and can be ignored; therefore:

(17) $$p^{\prime }\left(\vec{x},t\right)=p_{T}^{\prime }\left(\vec{x},t\right)+p_{L}^{\prime }\left(\vec{x},t\right)$$

## 3 Results

### 3.1 Grid independency

**Fig. 4:**
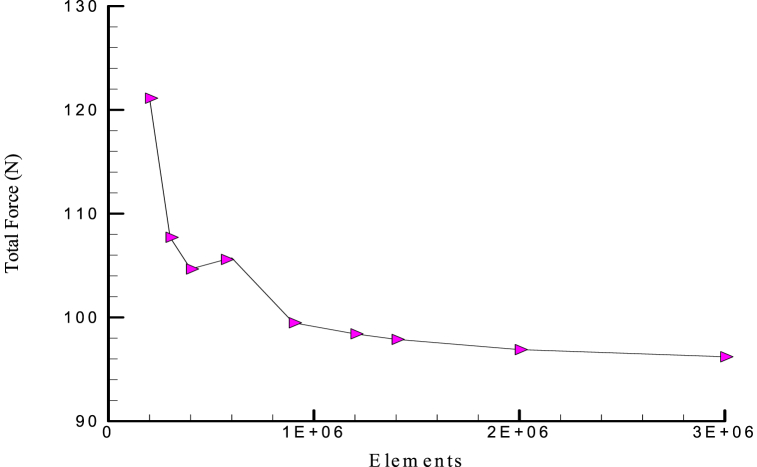
The independence of the total force results from the grid.

As can be observed from the grid independency study where the number of cells is more than 2 million, these forces are no longer dependent on the grid. So, it can be concluded that for a grid with 2016435 cells, the results have reached relative grid independency. Then, for the consistency of the results, a cell number of 2 million or more was used for all simulations (Fig. 4).

### 3.2 Validation

**Fig. 5:**
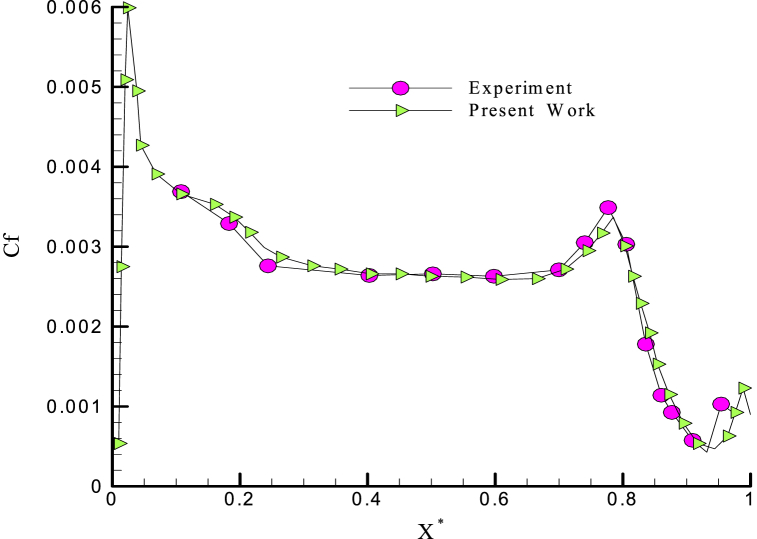
Comparison between the skin friction factor and the experimental results in Ref. [22].

The results obtained by the selected mesh are compared with the experimental results (Fig. 5) in Refs. [22,23].

**Fig. 6:**
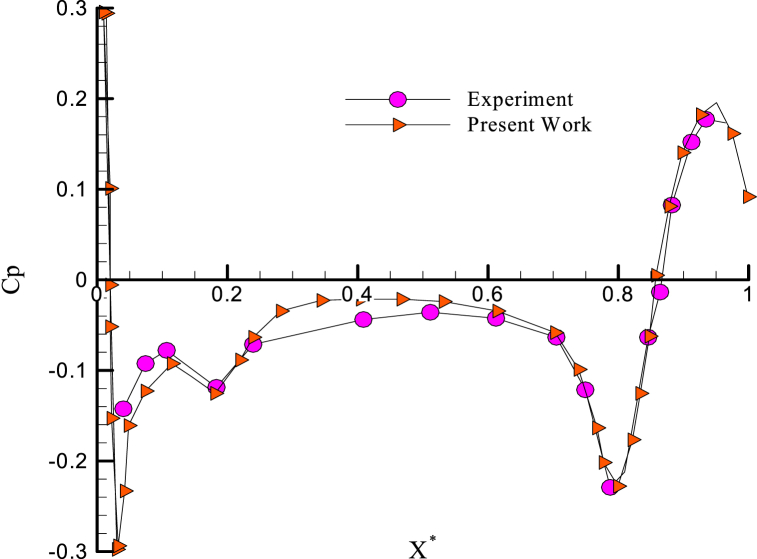
Comparison between pressure coefficient with the experimental results of Ref. [22].

There are experimental results in the literature for the different Reynolds numbers and the angle of attack zero [16,17]. Therefore, the results are compared with the experimental results in the same conditions (Fig. 6).

### 3.3 Some important factors in vessel profile design

The simple hull (bare) is the vessel's outer hydrodynamic form, including the pressure hull. To better judge and choose the best form of a simple hull, the most important factors in designing a simple hull form include.

• Minimum resistance to immersion: aspect ratio (L/D) and heel and node shapes are important. Jerome et al. [24] studied the optimization of the submarine shape to the logical algorithm based on minimum resistance. The optimization was studied based on the minimum resistance at the depth of the breathing tube by Volker [23].
• General arrangement necessities
• The sufficient volume to provide enough buoyancy due to the given weight
• Minimum noise, especially around the acoustic tracker and acoustic sensors
• Minimum cavitation around the propeller
• Minimum propagated noise of the vessel foil
• The coefficient of “volume-water yield is an important parameter for choosing a good vessel form because both strength and volume parameters are considered. Bigger values of this coefficient represent better designs.

### 3.4 Investigating the effect of cylindrical part diameter

**Fig. 7:**
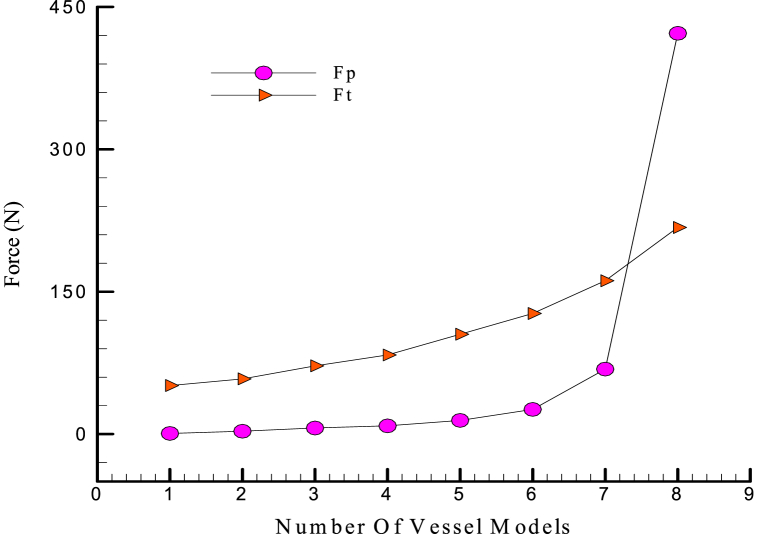
Comparison of compressive and frictional forces on different vessel models.

In Fig. 7, it is clear that the amount of frictional force is greater than the compressive force in all simulations, and this difference is still increasing in Model No. 6. But from Model 6 onwards, there is a sudden jump in the compressive force, and the dominant force in model 8 is the compressive force.

**Fig. 8:**
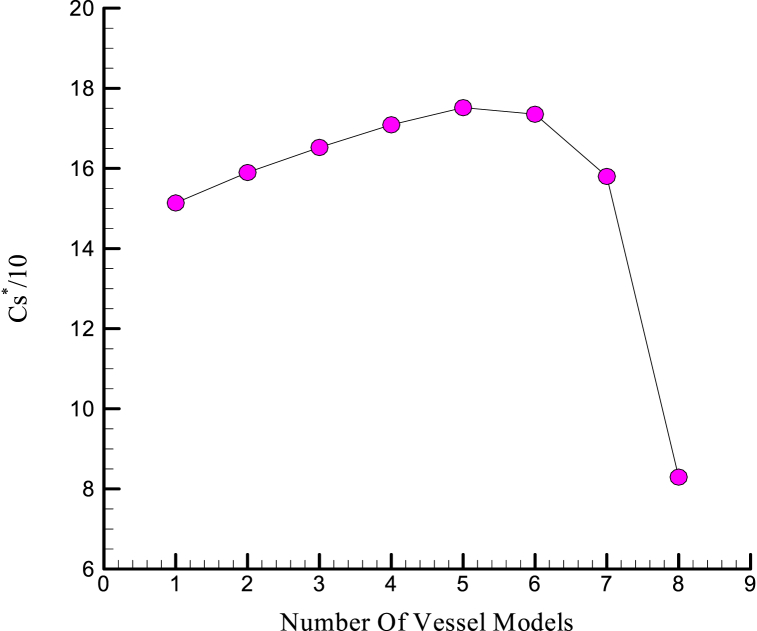
Modified volume factor correction charts for different vessel models.

The results of this change are shown in Fig. 8. According to the inferential results of the Semnan coefficient with the lower order, the modified Semnan coefficient curve in models 4 to 6 is maximum.

### 3.5 Study of the effect of nose and heel curves

**Fig. 9:**
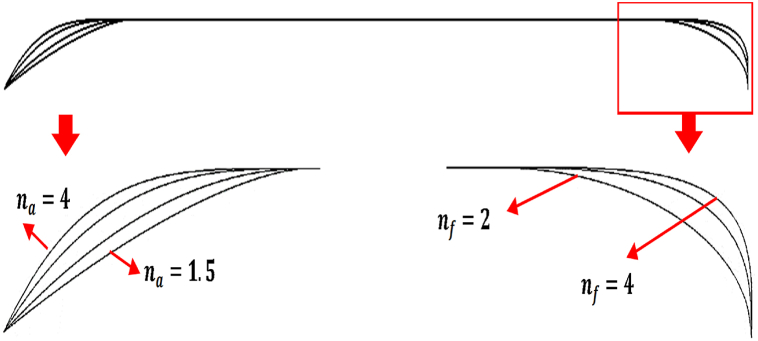
Different profiles of vessel nose and heel in one shape for better comparison of each item.

Here, different nose and heel profiles in the figure are presented to compare each item better. According to Fig. 9, increasing the nose shape becomes smoother, and $n_{a}$ the heel section becomes cylindrical.

**Fig. 10:**
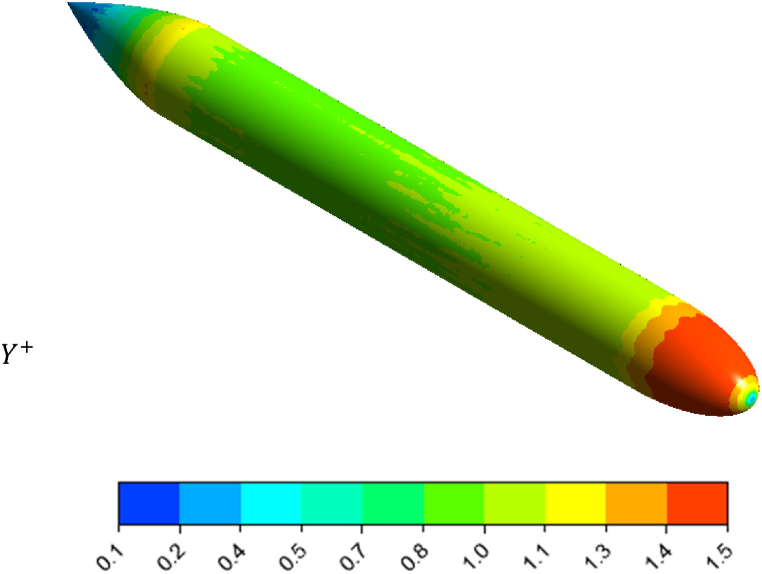
$Y^{+}$ Contour on the vessel hull surface with the values $n_{a}=1.5$ and $n_{f}=2$.

The value should be the first order to ensure the accuracy of the results and the correct simulation of the boundary layer. In Fig. 10, the $Y^{+}$ contour is plotted on the vessel hull surface with the values $n_{a}=1.5$ $n_{f}=2$.

**Fig. 11:**
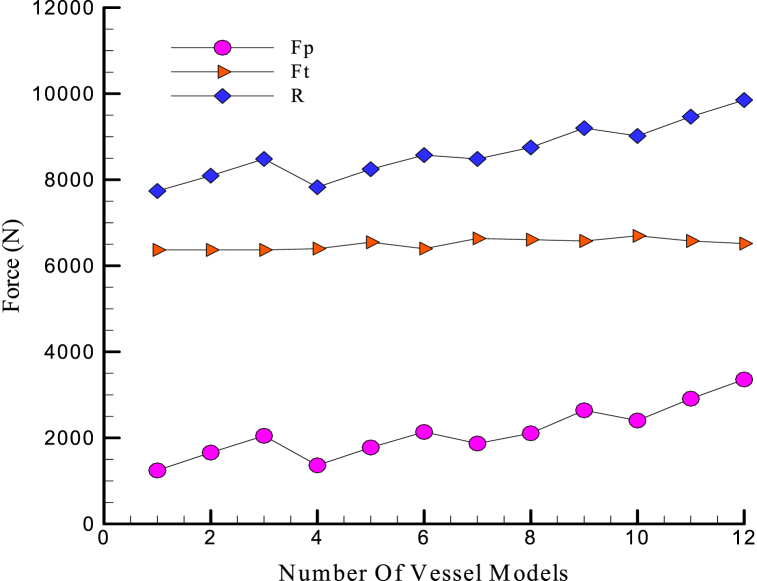
The values of compressive, friction, and resultant forces in terms of the model number.

If the values of compressive, friction, and resultant force are plotted in the model number, the following result is obtained in Fig. 11. The first point is that the frictional force values very insignificantly by increasing the model number and remains almost constant. However, as the model number increases, the compressive force has an increasing trend.

This means that the amount of frictional force that results from the viscosity of the water does not depend on the shape of the nose and heel and does not change with altering the curve shape of the nose or heel. In contrast, in analyzing the different hydrofoils of the SUBOFF model, this force is changed by varying the diameter of the cylinder part. Although the compressive force is less than the frictional force, the compressive force changes the resultant force. The compressive force plays a decisive role in the resistant force. Based on the graph, the process and form of the curve of the resultant force are the same as the compressive force curve and have the same extremum points. The frictional force only increases a constant value of the resultant force of each model. Sharper forms are better in volume return coefficient, while very sharp vessels are not desirable due to interior equipment placement and construction issues.

**Fig. 12:**
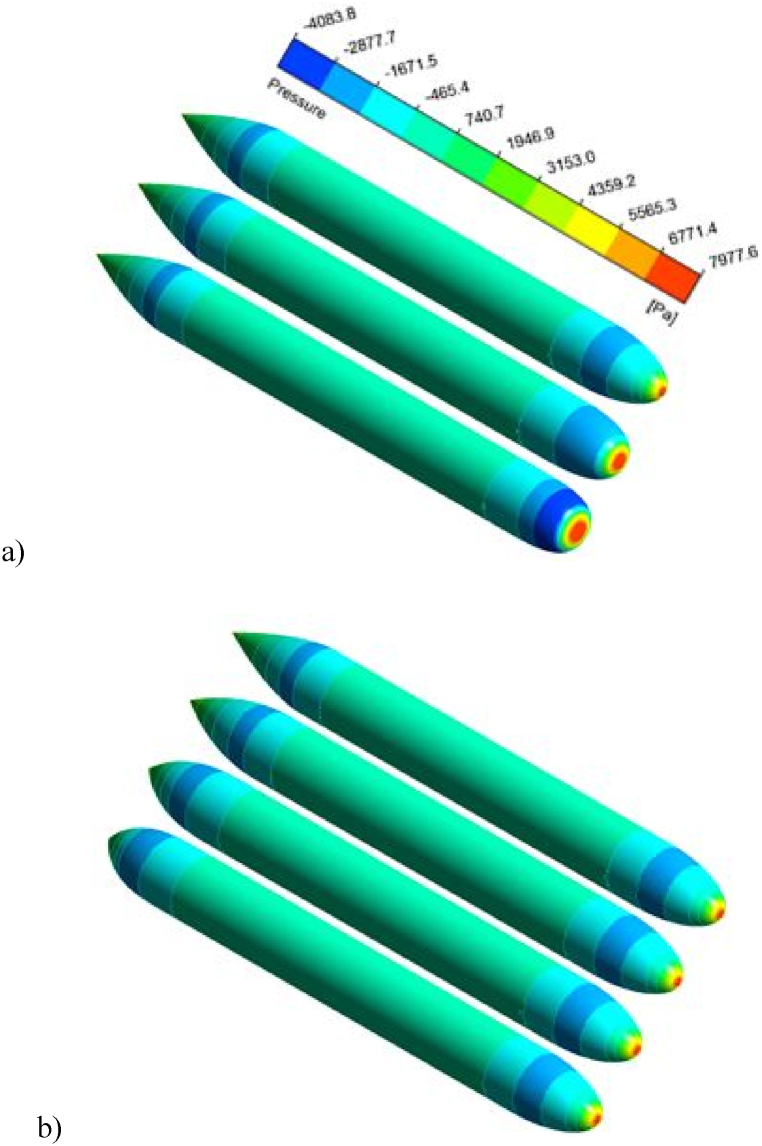
Three-dimensional pressure contour on the hull of undersurface vessel with different nose and heels.

**Fig. 13:**
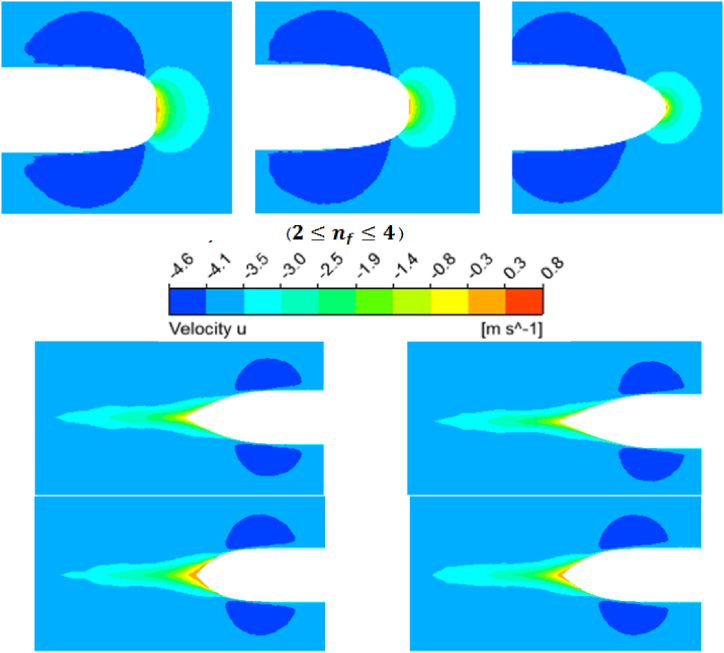
Velocity contour on the nose and heels of undersurface vessels.

**Fig. 14:**
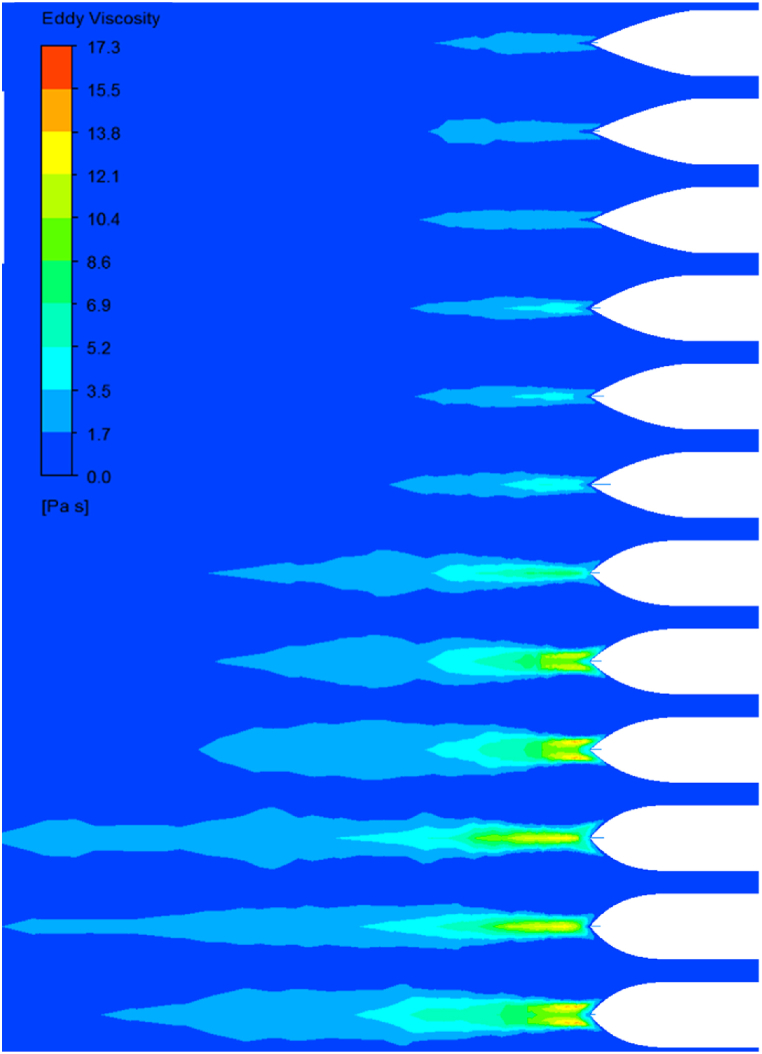
Comparison between the turbulence occurring in the vessel's wake for all simulated vessels in terms of the model number.

In Fig. 12, the 3D pressure contour is obtained on the hull of the undersurface vessel with different noses and heels. In Fig. 12 (a), three different noses with the same heel are compared. When the nose is flat, the pressure at the tip of the nose is increased on the vessel hull. On the other hand, the negative pressure in the nose area decreases, or the pressure difference between the high and low pressure (or negative pressure) regions increases. Section (b) obtains the pressure contour on the vessel hull with different heels and the same nose. As the $n_{a}$ increases, the heel becomes more similar to the cylindrical part. Hence, the slope of the final curve increases and causes a further decrease in pressure and an increase in areas with negative pressure. This reduction in the pressure on the heel increases the pressure gradient and subsequently increases the compressive force applied to the vessel hull. In the next Fig, the velocity contour is plotted on the nose and heels of simulated vessels. As $n_{f}$ it increases, the nose takes its flattened position, which increases the regions with zero velocities or, in other words, the fixed points. Reducing velocity in fixed points will increase the pressure. In Fig. 13, the velocity contour was investigated around the vessel hull on four heels. Increasing $n_{a}$ raises the curvature of the heel end, which increases the separation of flow and return flow. The backflow phenomenon causes the eddy currents to increase, which causes a loss of kinetic energy, which plays a significant role in increasing the drag and turbulence at the vessel's wake. In Fig. 14, the turbulence in the vessel wake is simulated for all vessels, and the model number is compared. As the model number increases, the turbulence rate increases. This means that it does not matter which constant increases, but increasing each constant $n_{a}$ and $n_{f}$ the turbulence increases steadily. The degree of turbulence caused by this is important because, in some cases, the use of such vessels requires moving in secret mode. Therefore, the degree of turbulence can also be considered as one of the factors influencing the selection of a vessel's hydrofoil.

### 3.6 The results of two different hull noises in terms of distance from the receivers

For a more comprehensive examination, the effect of all amplitudes of the frequency spectrum should be investigated. Accordingly, the concept of OASPL is used, which is expressed by the following Eq. (6).

(18) $$p^{\prime }\left(\vec{x},t\right)=p_{T}^{\prime }\left(\vec{x},t\right)+p_{L}^{\prime }\left(\vec{x},t\right)$$

**Fig. 15:**
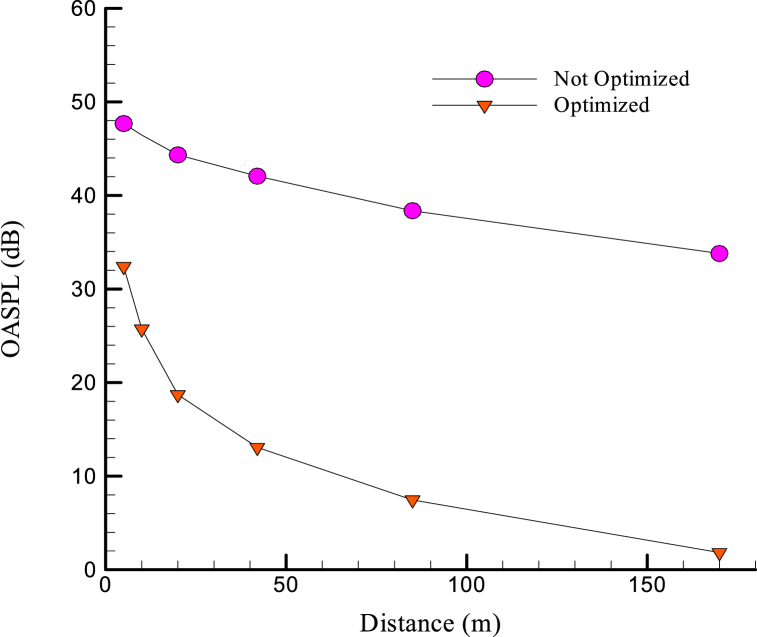
The results of noise in two different hulls in terms of distance from different receivers for a frequency of 2 kHz.

In fact, in the above equation, the compilation is made on all SPLs at the desired frequency range, provided that its effect in the OASPL number is minimized for the small or negative SPL values. The effect of negative SPLs that indicate the anacoustic zone can be ignored in the OASPL. In Fig. 15, the hydroacoustic results for two different hulls at a frequency of 2 kHz are calculated in different receivers. The results indicate the significant effect of the hull form on the hydro-acoustic noise of the hull. So, for the optimized hull, the fine-tuned noise ratio for all receivers is always less than the optimized model. It is observed that at far distances from the undersurface vessel for the optimized model, the OASPL value approaches the negative amounts indicating a near-an acoustic zone. Another interesting point is that the linear slope of the optimized hull is not optimized more than the hull. This means that the caused turbulence by the optimized hull has higher damping potential.

## 4 Conclusion

This paper investigates the shape of the container used and the resistance force and volumetric-aqueous efficiency using the CFD method. In this section, all the conclusions from the results section are summarized.

• After simulating the various profiles obtained from the SUBOFF equations, it was concluded that for models with less $R_{\max}$, most of the resultant force is caused by the frictional force, that is, the frictional force has a dominant effect, while with increasing $R_{\max}$ (or model number) the number of compressive force increases which continues until the point that the effect of the dominant force on the resultant force will be the effect of the compressive force.
• Increasing $n_{a}$ raises the curvature of the heel end, which increases the separation of flow and return flow. The backflow phenomenon causes the eddy currents to increase, which causes a loss of kinetic energy, which plays a significant role in increasing the drag and turbulence at the vessel's wake.
• By increasing the $n_{a}$ or $n_{f}$ constants, the turbulence increases steadily. The degree of turbulence is important because, in some cases, such vessels require moving in secret mode. Therefore, the degree of turbulence can also be considered as one of the factors influencing the selection of a vessel's hydrofoil.
• The results indicate the significant effect of the hull form on the hydro-acoustic noise of the hull. In other words, by optimizing the hydrodynamic form of the hull, the noise propagation can be reduced as much as possible.
• The linear slope of the optimized hull is not optimized more than the hull. This means that the caused turbulence by the optimized hull has higher damping potential.

## CRediT authorship contribution statement

**Zhiheng Xu:** Data curation, Conceptualization. **Yan Shi:** Supervision, Software. **Shelesh Krishna Saraswat:** Visualization, Validation. **Dheyaa J. Jasim:** Visualization. **Ahmad Keshavarzi:** Writing – review & editing, Writing – original draft. **Soheil Salahshour:** Visualization, Validation. **Ahmed Alawadi:** Writing – original draft, Methodology. **S.A. Eftekhari:** Data curation, Conceptualization.

## Declaration of competing interest

The authors declare that they have no known competing financial interests or personal relationships that could have appeared to influence the work reported in this paper.

## Abbreviation

CFD: Computational fluid dynamics
RANS: Reynolds-averaged Navier–Stokes equations
